# Prospecting for Genes involved in transcriptional regulation of plant defenses, a bioinformatics approach

**DOI:** 10.1186/1471-2229-11-88

**Published:** 2011-05-19

**Authors:** Marcel C van Verk, John F Bol, Huub JM Linthorst

**Affiliations:** 1 Institute of Biology, Leiden University, Sylvius Laboratory, Sylviusweg 72, 2333 BE Leiden, The Netherlands

**Keywords:** Co-expression analysis, salicylic acid-induced, jasmonic acid-induced, ethylene-induced, defense response, signal transduction, Arabidopsis, transcription factors

## Abstract

**Background:**

In order to comprehend the mechanisms of induced plant defense, knowledge of the biosynthesis and signaling pathways mediated by salicylic acid (SA), jasmonic acid (JA) and ethylene (ET) is essential. Potentially, many transcription factors could be involved in the regulation of these pathways, although finding them is a difficult endeavor. Here we report the use of publicly available Arabidopsis microarray datasets to generate gene co-expression networks.

**Results:**

Using 372 publicly available microarray data sets, a network was constructed in which Arabidopsis genes for known components of SA, JA and ET pathways together with the genes of over 1400 transcription factors were assayed for co-expression. After determining the Pearson Correlation Coefficient cutoff to obtain the most probable biologically relevant co-expressed genes, the resulting network confirmed the presence of many genes previously reported in literature to be relevant for stress responses and connections that fit current models of stress gene regulation, indicating the potential of our approach. In addition, the derived network suggested new candidate genes and associations that are potentially interesting for future research to further unravel their involvement in responses to stress.

**Conclusions:**

In this study large sets of stress related microarrays were used to reveal co-expression networks of transcription factors and signaling pathway components. These networks will benefit further characterization of the signal transduction pathways involved in plant defense.

## Background

Plants exposed to biotic and abiotic stress activate various signal transduction pathways, like the salicylic acid (SA)-, jasmonic acid (JA)-, ethylene (ET)-, and abscisic acid (ABA)-mediated signaling pathways that act singly or in combinations to evoke the most appropriate defense response [1-6]. For example, attack by pathogens results in extensive crosstalk between the SA-, JA- and ET-signaling pathways, implicating complex regulatory networks underlying the plant's pathogen defense [3]. Arabidopsis contains almost 1500 genes encoding transcription factors [7] and it is safe to assume that many are involved in regulation of these defense-signaling pathways. However, the precise regulatory mechanisms and the transcription factors involved are mostly still unknown. To fine-tune the initiated defense responses the biosynthesis and signaling pathways influence each other via crosstalk. This makes discovery of novel regulatory elements within these pathways even more challenging.

The signaling that leads to defense proceeds via interactions of signaling pathway components and because of this, the genes involved are often expressed under similar conditions. This makes their expression cooperatively regulated and their expression patterns highly similar. Based on this concept, an analysis of co-regulated genes under a variety of conditions can give valuable information for understanding the possible regulatory mechanisms involved in defense responses. Any dataset consisting of at least two experiments can be used to perform a co-expression analysis, although for an analysis that is independent of the experimental conditions, a minimum of approximately 100 experiments is needed [8].

To investigate co-expressed genes in Arabidopsis many co-expression databases from different micro-array sources with hundreds of experimental conditions per dataset have been developed in the last couple of years, such as Gene Expression Omnibus (http://www.ncbi.nlm.nih.gov/geo/[9]), ArrayExpress (http://www.ebi.ac.uk/microarray-as/ae/[10]), AthCor@CSB.DB (http://csbdb.mpimp-golm.mpg.de[11]), Genevestigator (http://www.genevestigator.com[12-14]), The Botany Array Resource (BAR; http://bbc.botany.utoronto.ca[15]), Arabidopsis Co-expression Data Mining Tool (ACT; http://www.arabidopsis.leeds.ac.uk/act/[16]), ATTED-II (http://atted.jp[17-19]), AtGenExpress/PRIME (http://prime.psc.riken.jp/[20]), and CressExpress (http://www.cressexpress.org[21]). Many of these databases only accept single-gene queries for analysis of a correlation coefficient. To obtain full flexibility in analysis method, data selection, filtering, etc., a more tailor made approach is needed. This can only be achieved after downloading the datasets and perform a manual analysis, which requires considerable computer power and knowledge about analysis methods, which is not essential for most of the available online tools.

Within the plant field there is an increasing number of publications that report the finding of biologically relevant genes involved in certain pathways via co-expression analysis. Examples are: genes involved in root development [22], genes involved in mitochondrial functions [23], clusters of genes involved in primary and secondary cell wall formation [24], Myb transcription factors responsible for initiation of aliphatic glucosinolate biosynthesis [25], and clusters of genes in a network related to cold stress and biochemical pathways [26]. In all these cases co-expression analysis assisted in building a network that linked unknown regulatory elements to already described pathways and helped expand hypotheses on how the genes in the network were regulated.

Although co-expression analysis tools are powerful in lead discovery, they cannot guarantee that observed co-expression of genes is biologically relevant. Further analysis using the 'classical' genomic and/or metabolomic approaches will still be necessary to confirm the involvement of the discovered genes. Despite this, co-expression analysis has proven itself as a very powerful tool in the discovery of new targets for analysis in pathways or networks of interest, as it can much more rapidly provide insight into potentially important network genes than random gain of function or loss of function approaches.

Here we report findings from a co-expression analysis covering a large number of microarray data sets derived from stress-induced Arabidopsis. In addition to genes already known to be involved in various stress-response pathways, a large number of new candidate genes were identified that potentially participate in regulation of stress-responses.

## Results and Discussion

### Public Microarray Data Selection

To discover new leads in the transcriptional regulation of the SA, JA and ET biosynthesis and signaling pathways under stress conditions an analysis of multiple transcriptome co-expression profiles was setup. For a flexible setup that is not limited to predefined settings, datasets or processing of samples, a dataset was downloaded from the TAIR website (ftp://ftp.arabidopsis.org/Microarrays/analyzed_data/). This dataset consists of 1436 Affymetrix Arabidopsis 25K arrays obtained from NASCArrays and AtGenExpress. All microarrays were normalized by TAIR using the robust multi-array method (RMA).

**Figure 1:**
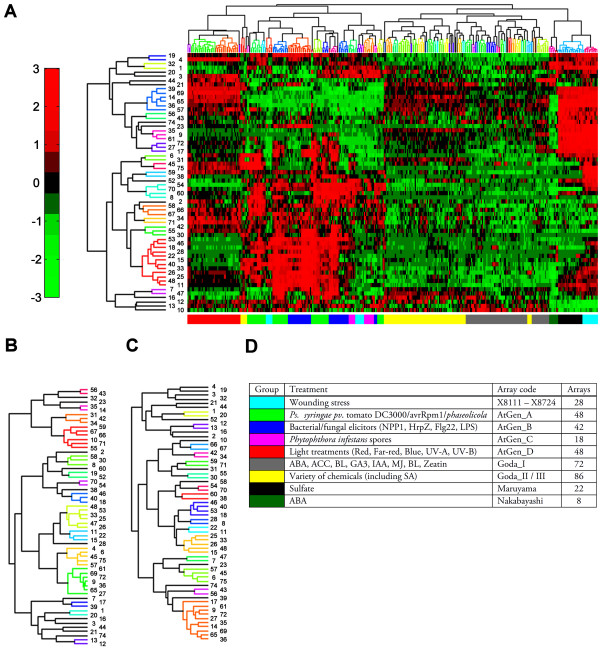
**Bi-clustering of WRKY genes under different experimental conditions**. Bi-clustering of WRKY genes under stress conditions (A), development-related processes (B), and all micro-arrays in the dataset (C). The colors in the bar underneath the bi-clustering in panel A correspond to the colored datasets of the selected microarray experiments listed in (D). The numbers on the left side of the bi-clustering indicate the corresponding WRKY numbers. Similarly colored branches within the dendogram represent groups with a linkage between nodes lower then 0.50. The color range in the bi-clustering matrix ranges from +3 (red, above average expression) to -3 (green, below average expression).

To focus on stress-related SA, JA and ET biosynthesis and signaling pathways we performed a bi-clustering of all WRKY transcription factors spotted on the Affymetrix arrays versus a selected set of microarray data obtained from a variety of stress conditions. The stress data set of 372 microarrays as listed in Figure 1D was selected from the total of 1436 currently available microarrays. An overview of these 372 microarrays is given in Additional file 1, Table 1. For comparison, a set consisting of 237 development-related microarrays and a set consisting of all 1436 available microarrays were also analyzed. Hierarchical cluster trees with complete linkage and dendogram cutoffs of 0.50 were added for both the experimental conditions and the WRKY genes, and visualized using different colors. The result of this bi-clustering is shown in Figure 1A. The colors of the bar below the bi-clustering matrix correspond to the colored sets of arrays as denoted in Figure 1D. Similar bi-clusterings of WRKY gene expression profiles were performed with the subset of development-related microarrays and with the set containing all micro-arrays. The hierarchical cluster trees for the latter bi-clusterings are shown in Figures 1B and 1C, respectively.

It is evident that substantial differences occur in the hierarchical clustering of the WRKYs between the three sets of arrays. WRKY genes with coordinated expression patterns clustering close together under conditions of stress (Figure 1A) appeared not necessarily also co-regulated during development (Figure 1B). E.g., WRKYs 19 and 4 (Figure 1A, top) were clustered close together in the same sub-tree when the bi-clustering was done with the set of stress microarrays, but were situated far apart in separate sub-trees when the development-related arrays were used. The same is the case for WRKYs 28 and 46 (see below). Therefore, to maximize the probability that only biologically relevant correlations were obtained, we chose to use the dataset of the stress-related microarrays listed in Figure 1D to investigate co-expression of genes involved in the SA, JA and ET pathways.

### Target Gene Selection and Co-expression Cutoff Determination

**Figure 2:**
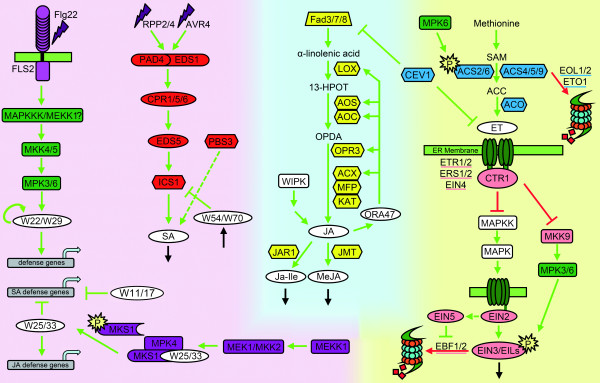
**Visual representation of the JA/SA/ET biosynthesis and signaling pathways**. Dark green boxes, MAPK kinases leading from flagellin to defense genes; red boxes, genes within the SA biosynthesis pathway; purple boxes, MAPK kinases leading to repression of SA and induction of JA defense genes; yellow boxes, genes involved in JA biosynthesis; light blue boxes, genes involved in ET biosynthesis; pink boxes, genes involved in ET signaling.

**Figure 3:**
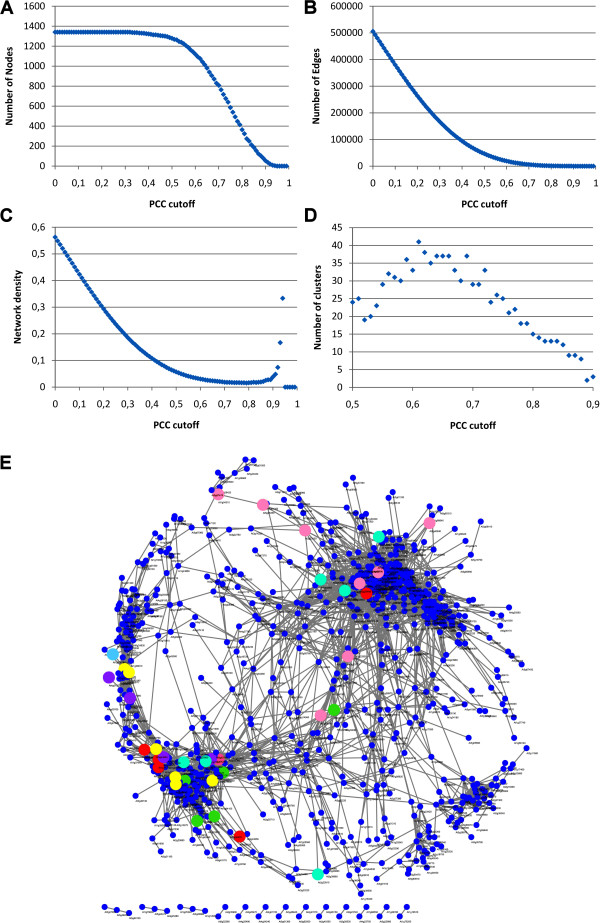
**Pearson correlation coefficient cutoff determination and co-expression network**. (A) Graph of the number of nodes with at least one link for each PCC cutoff. (B) Graph of the number of edges between nodes for each PCC cutoff. (C) Graph of the network density for each PCC cutoff. (D) Graph of the total number of clusters determined with the MCODE algorithm for each PCC cutoff. (E) Visualization using Cytoscape of the co-expression network. Blue-dots, on microarray spotted selection of >1400 transcription factors and JAZ proteins; other colored dots represent similarly colored genes from Figure 2.

To elucidate new transcription factors regulating SA, JA and ET biosynthesis and signaling pathways we composed a set of genes consisting of all color-coded genes indicated in Figure 2. This set comprises many well-documented genes attributed to the respective stress-signaling pathways [4]. This set was supplemented with a set of genes encoding almost 1400 transcription factors according to Czechowski *et al. *[7] and with the genes for the known JAZ repressor proteins and a number of other known regulators of these pathways. A listing of the genes in the set is given in Additional file 1, Table 1. To determine the Pearson Correlation Coefficient (PCC) cutoff for finding biologically relevant co-expressed genes and networks, various approaches can be applied. Several of these approaches are reviewed by Borate *et al. *[27] including maximal cliques, spectral graph clustering, correlation of control spots with expressed genes, top 1% of correlations, Bonferroni corrected p-values, and statistical power. The first two methods resulted in the most biological reliable PCC cutoffs. Since a maximal cliques approach required more computational power than we had available and the spectral graph clustering easily results in cutoffs that are 0.05 off, we chose to apply the approach as described by Aoki *et al. *[8]. Their method, based on density of the network combined with decreasing number of nodes and edges with higher PCC values, closely approaches the biological relevant PCC and is easy to implement for biologists with modest computing power. The number of nodes (genes), edges (links between genes), the network density (a ratio of the observed number of edges to all possible edges), and the number of individual clusters obtained using the MCODE algorithm was determined for different PCC cutoffs using the genes listed in Additional file 1, Table 1 and Figure 2. The results are visualized in Figure 3A-D. The total number of nodes and edges increased with a decreasing PCC threshold (Figure 3A and 3B). In Figure 3A a linear increase in the number of nodes that have at least one link with another node is found between 0.62 and 0.82. On the other hand, the number of edges below a cutoff of 0.70 starts to rapidly increase (Figure 3B), indicating that the available nodes become more densely connected as can also be seen with the increase in network density below this cutoff (Figure 3C). The region from 0.70 to 0.85 in Figure 3C indicates the minimum network density. According to the analysis of Aoki *et al. *[8] the most biological relevant PCC cutoff is found above these values. Combined, the data of Figure 3A-C leaves a relevant range for the cutoff between 0.70 to 0.82. To evaluate the number of clusters related to this range of closely co-regulated genes inside the network, the MCODE algorithm was used to determine the number of clusters for decreasing PCC values between 0.9 and 0.5 at 0.01 intervals (Figure 3D). The number of clusters increases steadily when lowering the PCC cutoff from 0.90 to approximately 0.70 after which it stabilizes between 0.72 and 0.60 and at lower thresholds even decreases. Combining the ranges of 0.60 to 0.72 and 0.70 to 0.82 made us choose the lowest overlapping cutoff of 0.70 for where biologically significant modules are most likely to be expected. We have not investigated networks of genes that are up-regulated in one set and down-regulated in the other (as would be represented by a negative PCC).

Using the PCC threshold of 0.70 a co-expression network was constructed and visualized with Cytoscape (Figure 3E). The blue dots represent the selection of transcription factors and JAZ proteins having at least one edge (i.e. sharing at least one connection with other genes), and the colored dots represent the correspondingly colored genes from Figure 2. The total co-expression network thus obtained consists of 808 nodes that share 5951 edges. Statistical verification of our choice of cutoff by calculation of Bonferroni corrected p-values cannot be applied with data sets of this size, since cutoffs of as little as 0.2 can easily become statistically highly significant, while biological relevance at this low cutoff would be unlikely [28]. However, close co-expression of genes as deduced from our constructed network matched well with correlations found in literature (see below). Moreover, biochemical and functional analysis with gene sets selected from our network further supported its robustness [29].

### Exploration of Co-expressed Closest Neighbor Transcription Factors of Regulatory Genes

**Figure 4:**
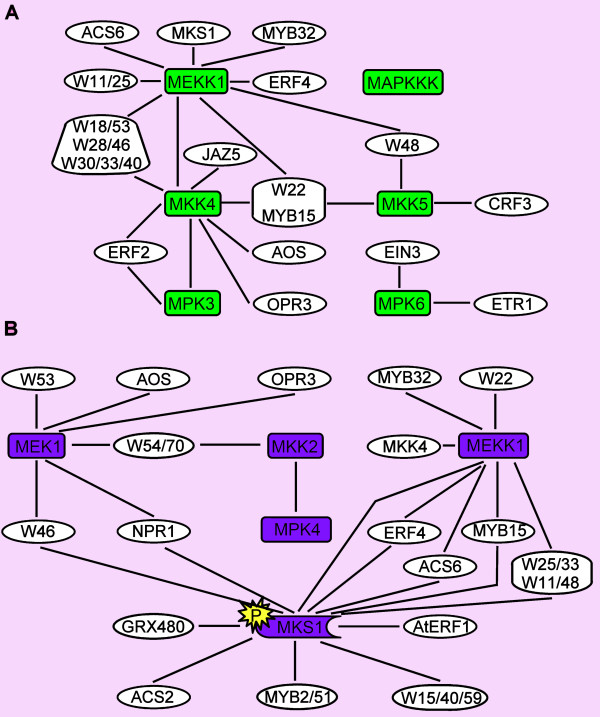
**Co-expression network of the MAP kinase pathways**. Co-expression network of MAP kinases leading to defense genes (A) and to SA defense gene repression and JA defense gene induction (B). The genes in colored boxes in the network correspond to similarly colored components of the signaling pathways indicated in Figure 2. The genes in white boxes indicate co-expressed genes with at least one edge to the kinase genes in the colored boxes.

**Figure 5:**
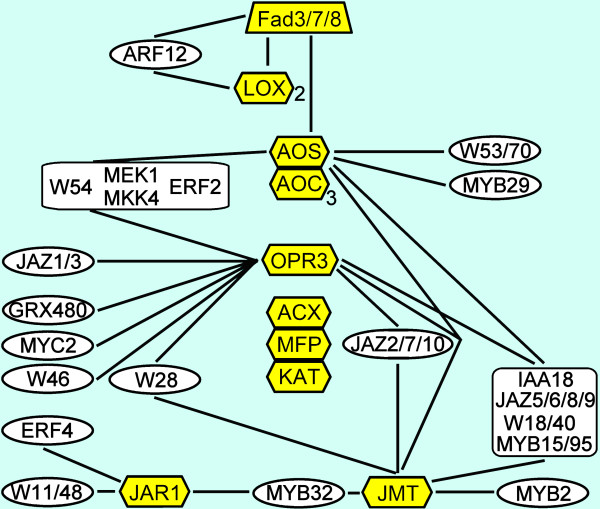
**Co-expression network of the JA biosynthesis pathway**. The genes in the yellow boxes in the network correspond to the yellow-colored components of the JA biosynthesis pathway indicated in Figure 2. The genes in white boxes indicate co-expressed genes with at least one edge to the pathway genes.

**Figure 6:**
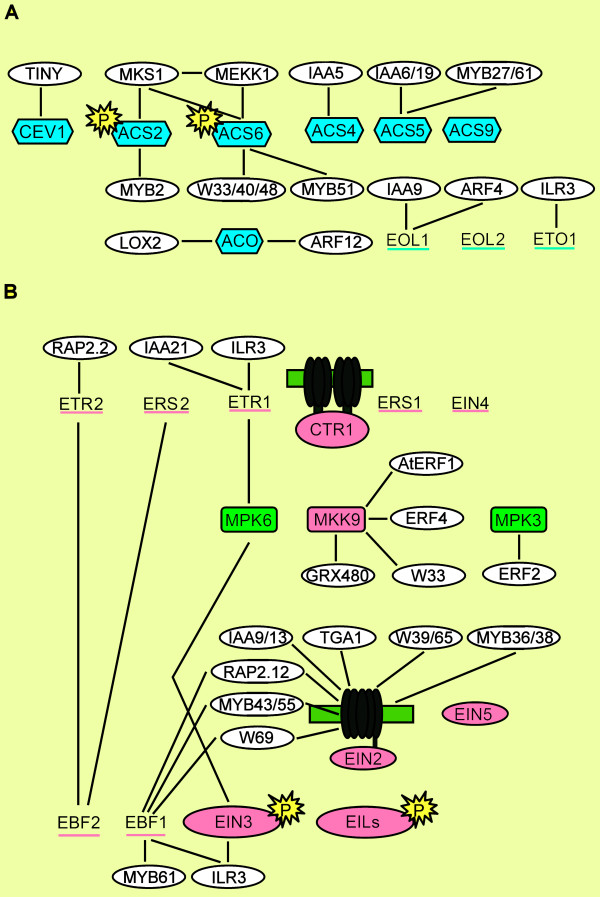
**Co-expression network of the ET biosynthesis and signaling pathways**. In panel A, the genes in the blue boxes in the network correspond to the blue-colored components of the ET biosynthesis pathway indicated in Figure 2. In panel B, The genes in colored boxes correspond to genes in similarly colored boxes of the ET signal transduction pathway shown in Figure 2. The genes in the white boxes in both panels indicate co-expressed genes having at least one edge to the pathway genes.

**Figure 7:**
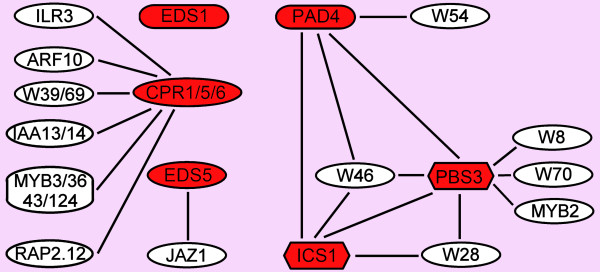
**Co-expression network of the SA biosynthesis pathway**. The genes in the red boxes in the network correspond to the red-colored components of the SA biosynthesis pathway indicated in Figure 2. The genes in white boxes indicate co-expressed genes with at least one edge to the pathway genes.

The closest neighbors with a single edge distance from the regulatory genes shown in Figure 2 were separated in multiple sub cluster networks (Figures 4, 5, 6 and 7). The MAP kinase pathway from flagellin to defense genes (Figure 2, dark green boxes) is depicted in Figure 4A, and the MAP kinase pathway leading to the suppression of SA and induction of JA defense genes (Figure 2, purple boxes) is shown in Figure 4B. The network of genes co-expressed with the JA biosynthesis genes (Figure 2, yellow boxes) is depicted in Figure 5. Networks of ET biosynthesis (Figure 2, light blue hexagons) and ET signaling (Figure 2, pink ovals) are shown in Figures 6A and 6B, respectively. Figure 7 shows the network of genes co-expressed with the genes leading to SA biosynthesis (Figure 2, red boxes). A detailed description of the above networks is given in the following paragraphs.

### The MAP Kinase Pathways

The response to flagellin fragment flg22 as part of the PAMP signaling pathway is mediated via a MAPK cascade [30,31]. This signal transduction via MAPKKK/MEKK1?-MKK4/MKK5-MPK3/MPK6 leads to transcriptional activation of downstream *WRKY22 *and *WRKY29 *genes, which results in the induction of resistance to both bacterial and fungal pathogens (Figure 2; [30]). Our results show that the genes encoding the MAPK components are highly co-expressed and form a network with a large number of co-expressed transcription factors (Figure 4A). The known downstream target of this cascade, *WRKY22*, is connected to *MEKK1 *and *MKK4*/*MKK5*. Surprisingly, *MPK6 *was not linked to any of the genes in the network, but appeared to be co-expressed with *EIN3 *and *ETR1*, both involved in the ET signaling pathway (Figure 4A; see below). As revealed by [32], multiple different models are possible of how MPK6 could be regulated directly under MEKK1. On the other hand, MPK6 has been described as the MAP kinase substrate of MKK3 and the MKK3-MPK6 cascade is important for the JA-dependent negative regulation of *MYC2*[33]. MYC2 has the opposite effect on the MKK4/MPK3 branch. Induction of *ERF2 *activates a variety of wound response/insect resistance genes in JA-treated plants and regulates JA-dependent responses. *ERF2 *is positively regulated by MYC2 and in our analysis is connected to *MKK4 *and *MPK3*[34,35]. Besides this connection, *MKK4 *is co-expressed with *AOS *and *OPR3 *(Figure 5) that are both important genes in the biosynthesis pathway of JA, suggesting that ERF2 might activate the MKK4/MPK3 cascade and via this route induce JA biosynthesis. With the biosynthesis of JA, in many cases also the JAZ repressor genes are positively regulated [36]. The connection between *MKK4 *and *JAZ5 *might indicate that this branch is under control of the JAZ5 repressor.

The flagellin fragment flg22 not only affects the regulation of JA and ET pathways, but also activates the SA pathway. Many WRKY genes are co-expressed with *MEKK1 *and *MKK4*. *WRKY28 *is rapidly induced to very high levels upon flg22 treatment [37]. Together with *WRKY28*, *WRKY46 *is also co-regulated and they are both found as co-expressed genes with important genes in the SA biosynthesis pathway (Figure 7).

Both WRKY18 and WRKY53 are positive regulators of *PR*-gene expression and systemic acquired resistance (SAR). Functional WRKY18 is required for full induction of SAR and is linked to the activation of *PR-1 *[38]. WRKY18 enhances resistance against *Pseudomonas syringae *[39]. The link between *WRKY53 *and *MEK1 *can be explained via *MEKK1 *(Figure 4B). MEKK1 is upstream of MEK1 and interacts with an activation domain protein that can be phosphorylated and binds to the promoter of *WRKY53* to activate gene expression [40]. This links *WRKY18 *and *WRKY53 *to flg22 and the initiation of SAR mediated defense within our co-expression network.

The MAPK cascade (MEKK1-MEK1/MKK2-MPK4), induced by challenge inoculation with *P. syringae *or treatment with flg22, leads to phosphorylation of MAP kinase substrate 1 (MKS1), which forms a complex with MPK4 and WRKY33 and possibly WRKY25. Upon phosphorylation of MKS1, WRKY33 is released inthe nucleus to initiate positive regulation of JA-induced defense genes and negative regulation of SA-related defense genes. Also other WRKYs, like WRKY11 and WRKY17, act as negative regulators of basal resistance responses [41-44]. Almost all of the genes encoding these WRKYs were found interconnected in the co-expression network (Figure 4B). *WRKY48 *is also stress and pathogen inducible and acts as a transcription factor that represses basal defense and *PR*-gene expression. When considering its location in the co-expression network, WRKY48 could function in a similar manner as WRKY11/17 and/or WRKY25/33 [45].

WRKY70 and the functional homolog WRKY54 have dual roles in SA-mediated gene expression and resistance. Upon high accumulation of SA, WRKY54/70 act as negative regulators of SA biosynthesis. Besides this negative role, they activate other SA-regulated genes [38,46]. The route via which WRKY54 and WRKY70 repress SA biosynthesis is unknown. Within the co-expression network both these WRKYs link to both *MEK1 *and *MKK2*, two important kinases in the cascade that leads to repression of SA defense genes. It may be that negative regulation of SA biosynthesis is brought about by activation of this MAP kinase cascade by WRKY54 and WRKY70.

### The JA Biosynthesis Pathway

The JAZ repressor proteins play an important role in JA signaling. The initial JAZ repressor that is released from MYC2 to activate transcription of target genes is JAZ3 [36,47]. *MYC2*, *JAZ1 *and *JAZ3 *are directly linked in the co-expression network with *OPR3*, encoding 12-oxophytodienoate reductase, an essential enzyme in JA biosynthesis (Figure 5). Several other genes encoding JAZ repressors are also connected to *OPR3 *and to the gene encoding *JA methyl transferase *(*JMT*), while others link to both *JMT *and the gene for *allene oxide synthase *(*AOS*). The various connections of these *JAZ *genes may hint at which levels the different JAZ repressors are operational (Figure 5).

Surprisingly, many of the WRKY transcription factors that are involved in positive or negative regulation of *PR*-genes and SAR are also connected to the JA biosynthesis pathway (Figure 5), like the positive regulatory combinations *WRKY18*/*53 *(Figure 4A), *WRKY54*/*70 *(Figure 4B), *WRKY28*/*46 *that are possibly involved in the regulation of SA biosynthesis (Figure 7) and *WRKY11/48 *that act as negative regulators of SA defense genes.

Several members of the MYB transcription factor family were also found to be closely co-expressed with the JA biosynthesis genes *AOS*, *OPR3 *and *JMT*. Most of the co-expressed MYB transcription factors have no known function. Using publicly available online co-expression analyses, a link was found between *MYB29 *and the regulation of aliphatic glucosinolate biosynthesis [25]. Since methyl-JA is involved in regulation of glucosinolate biosynthesis this could indicate that *MYB29 *is co-expressed at the level of *JMT *or below. However, the upstream connection of *MYB29 *with *AOS *suggests that activation of the glucosinolate pathway by MYB29 is already initiated before methyl-JA is synthesized.

### The ET Biosynthesis and Signaling Pathway

ET is produced from S-adenosyl-methionine in a two-step reaction catalyzed by the enzymes aminocyclopropane carboxylic acid (ACC)-synthase (encoded by *ACS *genes) and ACC-oxidase (encoded by *ACO*), respectively. Genes co-expressed with the ET biosynthesis genes are depicted in Figure 6A. We found a connection between *ACS2/6 *and *MEKK1/MKS1 *of the MAP kinase pathway. MEKK1 has been proposed to lead to phosphorylation of MPK6, although the mechanism through which this might occur has not yet been established. Different models for this regulation have been proposed [32]. ACS2 and ACS6 can be phosphorylated by MPK6 (Figure 2). This phosphorylation stabilizes the protein, which results in increased ET production [48]. Other genes co-expressed with the ET biosynthesis genes *ACS4*, *ACS5 *and *ACO *encode a variety of Aux/IAA and ARF factors. In a review by Reed [49] it is proposed that targets of Aux/IAA and ARF might include genes encoding ACC synthase. Various other Aux/IAA and ARF genes were found to be closely co-expressed with a number of other regulator genes (encoding ubiquitin ligases *EOL1*, *ETO1*) involved in ET biosynthesis, indicative of a possible function in the integration of ET and auxin signaling pathways.

The MAP kinases in the ET signaling pathway (Figure 6B) are connected to a limited number of other nodes. The link between *MPK3 *and *ERF2 *was discussed above. Mutant studies with the *etr1-1 *gain-of-function ET-insensitive mutant placed MPK6 directly downstream of ETR1 [50,51]. This is also observed within the co-expression network. In the network *EIN3 *is also connected to *MPK6*. In the MKK9-MPK3/6 cascade it was shown that direct phosphorylation in the nucleus via this cascade stabilizes the EIN3 protein, which may be a key step in ET signaling (Figure 2; [52]). The involvement of MKK9 at this point of the pathway has recently been questioned [53]. Notably, in the co-expression network MKK9 doesn't correlate with any genes known to be involved at this point of the pathway, further undercutting the suggested involvement of MKK9 in ET-signaling. Within the co-expression network depicted in Figure 3E both genes for *ETR1 *and *MPK6 *(represented by the pink and green dot almost in the middle of the network), are in between the super cluster with the genes encoding proteins involved in SA signaling (red dots), Flg22-initiated MAPK kinase cascade (green dots) and the JA biosynthesis genes (yellow dots), and the super cluster with several genes involved in the ET signaling pathway (pink dots). The central location of *MPK6 *and *ETR1 *between the super clusters with the other signaling genes might be indicative for a role of the combination of ETR1/MPK6 in crosstalk between these clusters.

Within the ethylene-signaling network (Figure 6B) we found many genes co-expressed with *EIN2*. For almost none of these genes a clear function has been described in literature so far. Recently, it was found that the modulation of NPR1 dependency of SA-JA crosstalk by ET is dependent on *EIN2 *[54]. Most of the genes involved in the crosstalk have not yet been assigned to a particular function. Surprisingly, in our analysis many of the genes that are co-expressed with *EIN2 *(*IAA13*, *RAP2.12*, *MYB36*, *MYB43*, *WRKY39*, *WRKY69*) are also connected to *CPR5 *in the SA biosynthesis pathway (see below). It is tempting to assume that some of these genes are involved in the *EIN2*-dependent crosstalk with SA.

### The SA Biosynthesis Pathway

Heterodimerization of EDS1 and PAD4 and their nuclear localization are important for subsequent steps in the SA signaling pathway [55]. Recently, it was found that *EDS1 *expression is repressed by the Ca^2+^/calmodulin-binding transcription factor AtSR1, indicating that SA levels are regulated by Ca^2+ ^[56]. We found that the gene encoding the Ca^2+^/calmodulin-binding transcription factor MYB2, is co-expressed with *PBS3 *(Figure 7; [57]). If MYB2, like AtSR1, acts as a repressor of SA accumulation, this might indicate another point of regulation. In addition to the link to *PBS3*, *MYB2 *is also connected to *JMT *in the methyl-JA synthesis pathway and to *ACS2 *in the ET biosynthesis pathway, suggestive for a role of MYB2 in fine-tuning SA, JA, and ET biosynthesis. Besides the connections of *WRKY54 *and *WRKY70 *that are already known to influence biosynthesis of SA, we found two new WRKY genes (*WRKY28 *and *WRKY46*) to be co-expressed with *isochorismate synthase 1 *(*ICS1*), a key enzyme in the biosynthesis of SA. As described above, *WRKY28* is known to be rapidly induced by flg22, while *WRKY46 *is rapidly induced downstream of avirulence effectors [58]. This might indicate a direct role for these WRKYs in flagellin and avirulence effector-induced biosynthesis of SA. Another *WRKY* gene that we found to be co-expressed with *PBS3 *is *WRKY8*. This WRKY is described in literature as one that is evolutionary highly related to WRKY28 [59].

**Figure 8:**
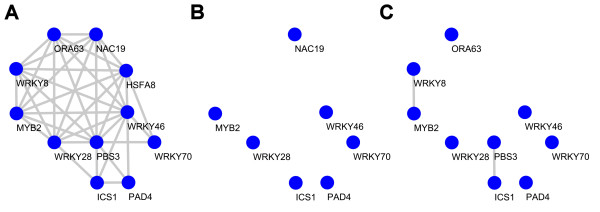
**Co-expression subnetworks of *ICS1 *and *PBS3***. The sub-network of genes that are co-expressed within one edge of *ICS1 *and *PBS3 *as obtained from the data sets of stress-related Arabidopsis microarrays (A), development-related microarrays (B), and all micro-arrays (C). Nodes from panel A are only shown in panels B and C if they have at least one edge within our outside of the *ICS1 *and *PBS3 *network.

To illustrate the validity of our choice to limit the co-expression analysis to the set of stress-related micro-arrays, in Figure 8 we focused on the sub network around *ICS1*/*PBS3*. In Figure 8A, all genes that were found co-expressed in the stress-related set within one edge at the PCC cutoff of 0.7 are displayed. Among the co-expressed genes are *WRKY70 *and *PAD4*, which are proven factors in the SA-signaling pathway [38,55]. This sub-network degraded when only the set of development-related genes (Figure 8B) or the set of all 1436 available micro-arrays were considered (Figure 8C). This supports the notion that also other genes in the dataset may play roles in the stress-related pathways investigated. Based on the results of the co-expression sub-network around *ICS1 *and *PBS3*, in a follow-up paper we investigated the possible role of transcription factors WRKY28 and WRKY46 in *ICS1 *and *PBS3 *gene expression [29].

In Figures 4, 5, 6 and 7 only co-expressed, established transcriptional regulators are depicted. A full list of all genes found to be closely co-expressed with the pathway components in Figure 2 is given in Additional file 2, Table 2.

## Conclusions

Our study shows that co-expression analysis using a selection of publicly available stress related data sets resulted in many new, potential components of the signal transduction pathways involved in stress responses. This could aid in the further characterization of these pathways.

## Methods

### Microarray Dataset

The dataset of 1436 Affymetrix Arabidopsis 25K arrays obtained from NASCArrays and AtGenExpress was downloaded from ftp.arabidopsis.org. This dataset has already been normalized using the robust multi-array method (RMA). For tracking down the experimental conditions of the different arrays we used the mapping file provided and with assistance from the curators of TAIR the codes were converted into matching experimental conditions that can be found on the website. Based on these experimental conditions selections were made of stress- and development-related datasets that were used in our experiments.

### Bi-clustering, Pearson Cutoff Determination and Co-expression Analysis

For the bi-clustering the raw RMA normalized expression values were transformed such, to obtain mean expression values of 0 and a standard deviation of 1 for all rows. Clustering of the data was performed using the following parameters: the distance between objects in the data matrix was one minus the sample correlation between points (treated as sequences of values), linkage was set to complete (furthest distance), and the cutoff within the dendogram for the hierarchical cluster tree was set to 0.50. All values below this cutoff were given a different color for both the experimental conditions and the genes.

To determine a biologically relevant Pearson correlation cutoff, the number of nodes and edges and the network density were determined using the raw RMA normalized expression values for different PCC cutoffs ranging from 0 to 1 at 0.01 intervals per data point using the 372 microarrays from the selected set of stress-related micro-arrays. The total number of clusters was determined using the MCODE algorithm within Cytoscape for PCC cutoffs from 0.5 to 0.9 at 0.01 intervals using the following settings: loops not included, degree cutoff = 2, Haircut on, fluff off, node score cutoff = 0.2, K-score = 2, Max depth = 100.

The co-expression network was built using the raw RMA normalized expression values with a PCC cutoff of 0.70 for the stress dataset and was visualized using Cytoscape using standard settings.

## Authors' contributions

MVV designed the study, carried out the analysis, helped in data interpretation, and made the draft of the manuscript. JFB and HL helped in data interpretation and edited the manuscript. All authors have given final approval for this version to be published.

## Supplementary Material

Additional file 1 **Table 1**. Lists of the 372 microarrays and the genes used for analysis in combination with Figure 2. Click here for file

Additional file 2 **Table 2**. Genes encoding transcriptional regulators closely co-expressed with signaling pathway genes. Click here for file
